# Wearable-Compatible Detection of Mild Cognitive Impairment Using Novel Features Based on Sleep Stage Dynamics

**DOI:** 10.3390/brainsci16060562

**Published:** 2026-05-26

**Authors:** Dhanushka Wijesinghe, Ivan T. Lima

**Affiliations:** Department of Electrical and Computer Engineering, North Dakota State University, Fargo, ND 58105, USA; dhanushka.wijesinghe@ndsu.edu

**Keywords:** Mild Cognitive Impairment (MCI), hypnogram analysis, sleep stage dynamics, sleep stage transitions, temporal sleep features, sleep fragmentation, machine learning, digital biomarkers, non-linear classification, feature engineering

## Abstract

**Highlights:**

**What are the main findings?**

- Novel hypnogram-derived sleep dynamics features enabled effective classification of Mild Cognitive Impairment using lightweight machine learning models.
- The RBF SVM classifier achieved the best performance with 78.7% balanced accuracy and 0.778 ROC AUC on the MASS SS1 dataset.

**What are the implications of the main findings?**

- Sleep stage transition dynamics may serve as non-invasive and physiologically interpretable biomarkers for early cognitive decline.
- The proposed framework demonstrates strong potential for future integration into wearable sleep monitoring systems for accessible MCI screening.

**Abstract:**

**Background:** Mild Cognitive Impairment (MCI) is an early stage of cognitive decline and a major risk factor for dementia, typically diagnosed using neuropsychological assessments such as memory and executive function tests. While EEG-based detection has been widely explored, most approaches rely on raw signal analysis and computationally intensive deep learning models. In contrast, wearable devices use indirect behavioral proxies (e.g., activity patterns or sleep–wake patterns), limiting diagnostic specificity. Although substantial clinical evidence indicates altered sleep architecture in MCI, the use of sleep stage dynamics for MCI classification remains largely unexplored. **Methods:** We propose a lightweight and physiologically interpretable framework using novel features based on hypnogram-derived sleep dynamics. The method was evaluated on the MASS SS1 dataset (36 healthy, 17 MCI subjects) using five classifiers—Logistic Regression, Random Forest, XGBoost, Linear SVM, and RBF SVM—with leave-one-subject-out validation and threshold optimization. **Results:** RBF SVM achieved the best performance (accuracy: 77.4%, balanced accuracy: 78.7%, sensitivity: 82.4%, specificity: 75.0%, ROC AUC: 0.778), followed by Random Forest (accuracy: 77.4%, balanced accuracy: 77.1%) and XGBoost (accuracy: 71.7%, balanced accuracy: 73.0%). **Conclusions:** This proof-of-concept study demonstrates that features extracted from sleep stage dynamics are effective, non-invasive, and interpretable biomarkers for early MCI detection, with strong potential for integration into wearable sleep monitoring systems.

## 1. Introduction

Mild Cognitive Impairment (MCI) is a clinical condition characterized by measurable cognitive decline that exceeds normal aging while largely preserving functional independence. It is widely regarded as an intermediate stage between normal aging and dementia, particularly Alzheimer’s disease [1,2]. Epidemiological studies estimate that approximately 12% to 18% of individuals aged 60 years or older are affected by MCI, highlighting its growing prevalence in aging populations [1]. Importantly, MCI is associated with an increased risk of progression to dementia, with annual conversion rates ranging from 10% to 15%, and nearly one-third of individuals progressing within five years [1]. Despite its clinical importance, awareness remains limited, with more than 80% of individuals reporting little familiarity with MCI [1,3]. These factors emphasize the need for reliable, scalable, and accessible methods for early detection and monitoring.

Current approaches for identifying MCI primarily rely on clinical assessments, neuroimaging techniques, and biological biomarkers. Clinical diagnosis is typically based on standardized cognitive evaluations such as the Mini-Mental State Examination (MMSE) and the Montreal Cognitive Assessment (MoCA), which assess memory, attention, and executive function [4,5]. Neuroimaging methods provide complementary insights into structural and functional brain alterations, including hippocampal atrophy observed in magnetic resonance imaging (MRI), disrupted connectivity in functional MRI, and amyloid-beta or tau deposition detected using positron emission tomography [6,7]. In addition, biological biomarkers, particularly cerebrospinal fluid measures of amyloid-beta and tau proteins, have demonstrated strong associations with underlying neuropathology [8]. While these approaches improve diagnostic accuracy, they are often costly, invasive, and require specialized infrastructure, limiting their feasibility for large-scale or continuous monitoring.

To address these limitations, electroencephalography (EEG)-based approaches have emerged as a promising alternative for automated MCI detection. EEG provides a non-invasive and cost-effective means of capturing neural dynamics with high temporal resolution. A growing body of work has identified electrophysiological biomarkers associated with cognitive decline, including alterations in spectral power, functional connectivity, and task-related responses [9]. Building on these findings, machine learning and deep learning techniques have been increasingly employed to enhance classification performance. For instance, Chaabene et al. proposed a transformer-based model to capture temporal dependencies in EEG signals [10], while Yin et al. utilized integrated spectral-temporal feature extraction combined with advanced classifiers for improved discrimination [11]. More recently, Geng et al. demonstrated the effectiveness of deep learning applied to sleep EEG signals for MCI detection [12].

In parallel, connectivity-driven and network-based approaches have further advanced the field. Kolahkaj and Zare developed a connectome-based deep learning framework using structural brain networks, while Kam et al. proposed models incorporating both static and dynamic functional connectivity for early MCI detection [13,14]. Despite these promising results, most EEG-based methods rely heavily on raw signal processing and complex model architectures, requiring extensive preprocessing, high computational resources, and multi-channel recordings. Moreover, many studies focus on awake or task-based EEG, which limits their applicability for continuous, real-world monitoring. These challenges highlight a critical gap between high-performance research models and practical, deployable systems.

Concurrently, wearable and at-home monitoring technologies have gained attention as scalable solutions for early detection of cognitive decline. Advances in digital health have enabled the use of smartphones and wearable devices to capture behavioral and functional changes associated with MCI [15,16]. These approaches include monitoring daily activity, speech behavior, and mobility patterns in real-world environments. For example, smartphone-based applications such as MemScreen have demonstrated the feasibility of detecting MCI through structured cognitive tasks performed at home [17]. However, these systems primarily rely on indirect behavioral indicators rather than direct measurements of brain function, and often represent digitized versions of traditional cognitive assessments.

Sleep offers a unique and underexplored physiological domain that bridges this gap between clinical relevance and practical deployment. Significant progress has been made in automated sleep stage classification, with deep and machine learning models such as DeepSleepNet, TinySleepNet, and SeqSleepNet achieving performance exceeding 90% agreement with expert annotations [18,19,20,21]. In addition, recent advances in wearable EEG technologies have enabled reliable overnight sleep monitoring outside clinical environments [22]. Importantly, clinical studies have shown that MCI is associated with measurable alterations in sleep architecture and dynamics, including disrupted sleep continuity, reduced slow-wave sleep, altered Rapid Eye Movement (REM) characteristics, and abnormal transitions between sleep stages [23,24]. These findings suggest that sleep stage dynamics encode physiologically meaningful signatures of early cognitive decline.

Motivated by these observations, we hypothesize that sleep stage sequences, represented as hypnograms, contain sufficient information to enable accurate MCI detection without relying on raw EEG signals. Instead of directly processing high-dimensional EEG data, we propose to leverage features derived from sleep stage dynamics, enabling a lightweight and interpretable classification framework. Such an approach naturally aligns with existing sleep monitoring systems, allowing seamless integration into a two-stage pipeline: sleep staging is first performed using established algorithms, followed by MCI risk assessment based on derived sleep dynamics.

This paradigm introduces a practical pathway toward real-world deployment. In a wearable setting, overnight sleep data can be automatically processed to generate sleep stage sequences, after which the proposed lightweight model can assess MCI risk. Individuals can then receive early warnings based on repeated observations, facilitating timely clinical evaluation while avoiding invasive or expensive procedures.

In this study, we propose a novel machine learning framework for MCI detection based on hypnogram-derived sleep stage dynamics. To the best of the authors’ knowledge, this is the first work to systematically extract and utilize hypnogram-based features within a machine learning framework for MCI identification. The primary contributions of this work are two fold. First, we derive and identify a set of features that capture sleep dynamics directly from hypnograms, including both conventional sleep architecture descriptors and underexplored representations of transition behavior and temporal evolution. While traditional features such as wake after sleep onset (WASO), sleep fragmentation, and number of awakenings have been widely used in prior studies—often derived from raw EEG or multimodal signals—the systematic formulation of transition dynamics and intra-night temporal variations as machine learning features remains relatively underexplored. It is important to note that these features do not aim to introduce new physiological phenomena; rather, they provide a structured and quantitative formulation of well-established sleep characteristics, such as sleep fragmentation, stage transition behavior, and temporal organization, in a manner that is suitable for machine learning–based analysis.

Second, we present the first study that integrates these hypnogram-derived features into a lightweight machine learning framework for MCI detection, enabling a practical and deployable solution suitable for wearable-based systems. The framework is evaluated using the MASS SS1 dataset, comprising polysomnography recordings from healthy and MCI subjects. Multiple machine learning models, including Logistic Regression, Random Forest, Extreme Gradient Boosting (XGBoost), Linear Support Vector Machine (SVM), and Radial Basis Function Support Vector Machine (RBF SVM), are systematically compared under a leave-one-subject-out (LOSO) validation scheme with threshold optimization. In this context, the novelty of the proposed approach lies in the representation and integration of hypnogram-derived sleep-stage dynamics into a unified feature space, rather than in the discovery of fundamentally new biological mechanisms. The results aim to demonstrate that sleep dynamics alone provide sufficient discriminatory power for MCI detection while maintaining model simplicity and efficiency.

It is important to distinguish between the hypothesis-driven and data-driven components of this study. The feature design is motivated by established physiological knowledge of sleep dynamics and their association with cognitive impairment. However, the selection and evaluation of the most informative features are performed in a data-driven manner within the machine learning framework.

The remainder of this paper is organized as follows. Section 2 describes the dataset, preprocessing steps, feature extraction methodology, and machine learning framework. Section 3 presents the experimental results and performance evaluation across different models. Section 4 discusses the findings and their implications for wearable-based cognitive screening. Section 5 outlines the limitations of the study. Finally, Section 6 concludes the paper and outlines directions for future work.

## 2. Materials and Methods

**Figure 1:**
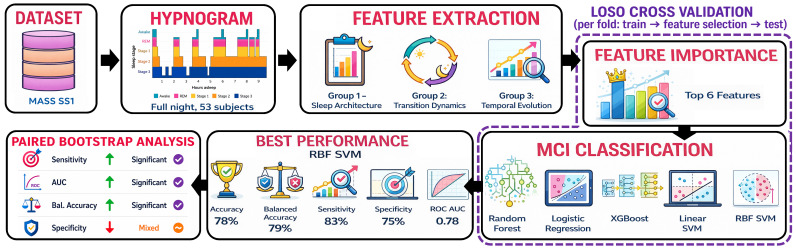
Overview of the proposed pipeline for MCI detection using hypnogram-derived sleep dynamics. Sleep stage annotations are converted into full-night hypnograms, from which features capturing sleep architecture, transition dynamics, and temporal evolution are extracted. Within each fold of the leave-one-subject-out (LOSO) cross-validation framework, feature importance analysis and model training are performed using training data only. Multiple machine learning models are evaluated, and paired bootstrap analysis is used to compare performance, demonstrating improvements in sensitivity, AUC, and balanced accuracy, with a corresponding trade-off in specificity.

An overview of the proposed framework for MCI detection using sleep stage dynamics is illustrated in Figure 1. At a high level, the methodology consists of extracting subject-level features from sleep stage annotations, followed by machine learning–based classification to distinguish between healthy and MCI subjects. The pipeline includes preprocessing of sleep stage sequences, computation of interpretable sleep architecture and transition-based features, and model training and evaluation using a leave-one-subject-out strategy. Each component of the framework is described in detail in the following subsections.

### 2.1. Dataset and Expert Sleep Stage Annotations

The analysis in this study is based on the MASS SS1 dataset, which includes overnight polysomnographic recordings from 53 subjects (age 63±5.3 years, range: 55–76 years), comprising 34 males (63.5±5.6 years) and 19 females (63.7±4.9 years) [25,26]. Sleep stages were scored by clinical experts according to the American Academy of Sleep Medicine (AASM) guidelines using 30-s non-overlapping epochs. Each epoch was assigned a single label from five classes: Wake, N1, N2, N3, or REM. The original MASS data collection was conducted under appropriate institutional ethics approval, with informed consent obtained from all participants by the original investigators. The present work represents a secondary analysis of previously collected de-identified data accessed in accordance with the MASS data usage conditions. Access to the restricted MCI identifiers was specifically approved by the MASS Scientific Committee.

In addition to sleep stage scoring, the dataset includes expert annotations such as apnea/hypopnea events and micro-arousals, as well as automatically detected events including muscular artifacts and periodic limb movements of sleep. However, these additional annotations were not utilized in the present study, as the focus is solely on sleep stage dynamics.

MCI labels were obtained from the associated clinical information, where 15 subjects were identified as MCI in the original dataset, along with 2 additional borderline cases diagnosed under updated clinical criteria. Since these borderline cases cannot be reliably separated, all 17 subjects were treated as MCI-positive in this work, which may introduce slight uncertainty in classification performance. Furthermore, epochs labeled as Not Classified (NC) were excluded prior to feature extraction to ensure consistency in the derived sleep stage sequences. Given the relatively small cohort size and the inclusion of borderline MCI cases, this labeling strategy may introduce a degree of uncertainty and potential label noise, which could influence model stability and affect generalizability.

### 2.2. Feature Extraction

The design of the proposed feature set is motivated by established findings in the literature linking both electrophysiological activity and macroscopic sleep structure to MCI. Prior studies have consistently reported that MCI is associated with alterations in brain oscillatory activity, particularly reflected in changes in power spectral density across canonical frequency bands such as delta, theta, and alpha [9]. These alterations suggest a disruption in neural synchrony and functional organization, indicating that sleep-related dynamics may serve as potential biomarkers of cognitive decline.

In addition to spectral abnormalities, several studies have highlighted significant changes in sleep architecture among individuals with MCI. Systematic analyses have reported increased sleep fragmentation, longer sleep latency, reduced sleep efficiency, and altered proportions of sleep stages, particularly reductions in deep sleep (N3) and REM sleep [23]. Furthermore, polysomnographic and self-reported sleep measures have shown that individuals with MCI tend to exhibit increased nocturnal awakenings, prolonged wake periods after sleep onset, and decreased overall sleep quality [24].

These findings collectively suggest that both micro-level neural activity and macro-level sleep organization are affected in MCI. To gain an initial qualitative understanding of these differences, subject-specific hypnograms were visually inspected. This inspection revealed that MCI subjects tend to exhibit increased sleep fragmentation, characterized by more frequent transitions between stages, such as from NREM sleep to wake, as well as noticeable alterations in the temporal organization of sleep across the night, including differences between early and late sleep periods.

**Figure 2:**
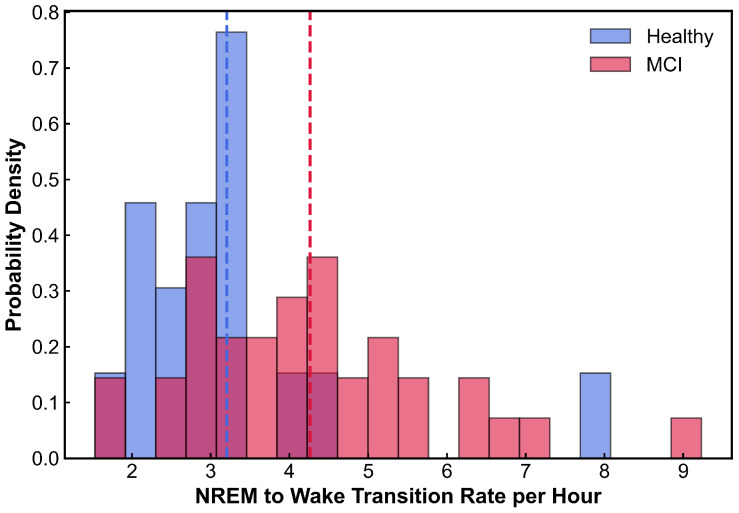
Distribution of NREM-to-wake transition rates per hour for healthy and MCI subjects, computed from expert-annotated sleep hypnograms in the MASS SS1 dataset. The MCI group exhibits a noticeable shift toward higher transition rates and a broader spread, indicating increased sleep fragmentation compared to healthy controls. This trend is consistent with previously reported alterations in sleep continuity in MCI. Vertical dashed lines denote the corresponding mean values for each group. While some overlap exists between the two groups, this trend suggests that sleep-stage transition dynamics may carry clinically relevant information related to cognitive impairment. This observation motivates the exploration of transition-based and temporal sleep features for MCI classification.

To systematically quantify these observations, we analyzed the distributions of several key transition-based sleep dynamics derived from the hypnograms. One representative example is shown in Figure 2, which illustrates the distribution of NREM-to-wake transition rates for healthy and MCI subjects. As can be observed, the MCI group exhibits a tendency toward higher transition rates and a broader distribution (see Section 2.2.2 for formal definitions), reflecting increased sleep fragmentation compared to healthy controls. This behavior is consistent with well-established findings on disrupted sleep continuity and increased fragmentation in MCI. Although this single measure alone is not sufficient to reliably distinguish MCI from healthy subjects, it highlights underlying differences in sleep dynamics. These observations motivate the formulation of a broader set of features that systematically capture transition behavior, temporal organization, and sleep-stage dynamics. When combined with a set of complementary features capturing sleep architecture, transition behavior, and temporal evolution, such information can be effectively leveraged for MCI classification. These observations therefore motivate the systematic development of a comprehensive feature framework for this task.

In this study, rather than directly analyzing raw EEG signals, we leverage sleep stage annotations to capture the macroscopic organization of sleep. These hypnogram-based representations provide a compact and physiologically meaningful summary of sleep dynamics while significantly reducing computational complexity. These hypnograms used by the algorithm for MCI classification can be extracted from raw EEG data processed by a machine learning algorithm as in [18,19,20,21].

Accordingly, the proposed feature set is organized into three complementary groups: (i) Sleep Architecture Features, which are primarily grounded in existing clinical and sleep research literature; (ii) Transition Dynamics Features, which introduce a structured representation of stage-to-stage evolution; and (iii) Temporal Evolution Features, which further extend this representation by capturing intra-night variations in sleep behavior. While Group 1 features provide a validated baseline, Groups 2 and 3 constitute the main methodological contributions of this work, not by introducing new physiological mechanisms, but by providing a systematic and quantitative formulation of well-established sleep characteristics. That formulation consists of combining fragmentation, transition behavior, and temporal organization into a unified feature space suitable for machine learning. Therefore, the novelty lies in the representation and integration of these dynamics rather than in the discovery of fundamentally new biological insights.

#### 2.2.1. Group 1: Sleep Architecture Features

The first feature group summarizes the overall macroscopic architecture of sleep. These features are well-established in the literature and are included to capture known alterations in sleep composition, continuity, and fragmentation associated with MCI. For each subject, the proportion of time spent in each canonical sleep stage (Wake, N1, N2, N3, and REM) was computed. A sleep efficiency proxy was defined as the fraction of valid scored epochs spent in sleep rather than wake.

To characterize temporal organization, sleep onset latency and REM latency were calculated as the time from the beginning of the recording to the first sleep epoch and the first REM epoch following sleep onset, respectively. Additional measures of sleep disruption were included, such as wake after sleep onset (WASO), defined as the total duration of wake epochs after sleep onset, and the number of awakenings per hour. To further quantify sleep continuity, the mean sleep run duration and mean wake run duration were computed based on consecutive stage segments. Finally, a stage entropy measure was included to capture the heterogeneity of the sleep-stage distribution. Collectively, these features provide a compact representation of sleep architecture consistent with prior clinical findings.

#### 2.2.2. Group 2: Sleep Transition Features (Proposed)

The second feature group introduces a novel set of descriptors designed to characterize the dynamic evolution of sleep stages. Unlike conventional approaches that primarily focus on stage proportions, this group explicitly models the temporal transitions between stages, enabling a deeper representation of sleep organization.

First, the transition rate per hour was computed as the number of stage changes between consecutive epochs normalized by the total duration, providing a measure of sleep instability. A first-order transition matrix was then constructed to capture the probabilities of transitioning between sleep stages. Let $N_{ij}$ denote the number of observed one-step transitions from stage *i* to stage *j*. The corresponding transition probability is given by

(1) $$P_{ij}=\frac{N_{ij}}{\sum_{k}N_{ik}},$$

where $P_{ij}$ is defined only when at least one outgoing transition from stage *i* is observed. The diagonal terms $P_{ii}$ represent self-transition probabilities and quantify stage persistence, while the off-diagonal terms describe preferred transition pathways between stages. To further characterize transition irregularity, the Shannon entropy of each transition row was computed as

(2) $$H_{i}=-\sum_{j}P_{ij}\log_{2}P_{ij},$$

and the mean row entropy across all valid stages was used as a global measure of transition irregularity.

These features collectively provide a quantitative and interpretable representation of sleep-stage dynamics, capturing persistence, switching behavior, and transition irregularity. Importantly, these descriptors are grounded in well-established physiological concepts such as sleep fragmentation and stage instability, and their contribution lies in formalizing these behaviors into a structured feature set suitable for machine learning. This transition-based modeling of sleep dynamics represents a key contribution of this study.

#### 2.2.3. Group 3: Temporal Evolution Features (Proposed)

The third feature group extends the proposed framework by capturing intra-night variations in sleep dynamics. While most prior studies consider sleep as a stationary process, this work explicitly models how sleep characteristics evolve over time, which is particularly relevant given that MCI has been associated with disrupted temporal organization of sleep.

To this end, each subject’s valid sleep stage sequence was divided into two equal segments representing the early and late portions of the night after excluding Not Classified (NC) epochs. For each segment, selected features from Groups 1 and 2 were recomputed independently, including stage proportions, fragmentation measures, transition rates, selected transition probabilities, and transition entropy. To explicitly quantify temporal variations, features were derived in four forms: (i) early-night features, (ii) late-night features, (iii) difference features defined as the late-night value minus the early-night value, and (iv) ratio features defined as the ratio of late-night to early-night values with a small regularization constant to ensure numerical stability.

These features enable the detection of deviations from normal sleep progression, such as reduced slow-wave sleep in the early night or altered REM distribution across the night. By incorporating both absolute and relative temporal changes, this group provides a complementary representation of sleep dynamics that captures temporal organization rather than introducing new physiological constructs.

Overall, the combination of Groups 2 and 3 establishes a unified feature space that captures both the dynamic and temporal aspects of sleep, enabling effective and lightweight MCI detection. A summary of all extracted features is provided in Table 1. While the formulation of these features is guided by prior knowledge of sleep physiology, their relative importance and contribution to MCI classification are determined through data-driven feature selection and model evaluation.

### 2.3. Feature Importance Analysis

To identify the most informative predictors for MCI detection, a feature importance analysis was performed using a RF framework within each LOSO cross-validation fold. In this approach, the RF model was trained on the training subset of each fold, and feature importance scores were computed based on each feature’s contribution to impurity reduction. Given the limited sample size of the dataset (53 subjects), careful consideration was given to controlling model complexity and avoiding overfitting. Therefore, instead of using the full feature set, reduced subsets consisting of the top 6, 8, and 10 most important features were evaluated within each fold, ensuring that only the most relevant and robust predictors were retained.

Subsequent model development and grid search analysis across multiple machine learning classifiers demonstrated that a compact subset of the top 6 features consistently achieved comparable or superior performance relative to larger feature sets. This observation indicates that a small number of well-selected features are sufficient to capture the key differences in sleep dynamics associated with MCI, while also enhancing model generalizability. Based on these findings, the final models were developed using the top 6 most important features identified within each LOSO fold. This design minimizes the risk of data leakage by ensuring that feature selection is performed within each training fold. However, given the limited dataset size and the repeated use of the same cohort for feature selection, hyperparameter tuning, and evaluation, there remains a possibility of implicit overfitting that is not fully captured by internal validation. The selected features and their physiological relevance are presented and discussed in detail in the Section 3.

### 2.4. Machine Learning Models

To evaluate the effectiveness of the proposed feature set, five supervised machine learning models were considered: LR, RF, Linear SVM, RBF SVM, and XGBoost. These models were selected based on the considerations outlined in this study, as well as their demonstrated effectiveness in previously published research on MCI detection and related biomedical classification tasks [27,28,29,30]. They are also well-suited for small datasets and provide complementary modeling capabilities. For each model, hyperparameters were optimized using a grid search procedure within the training data of each LOSO fold to ensure fair and unbiased evaluation. The key hyperparameters considered for each model are summarized in Table 2.

Prior to model training, feature standardization was applied to ensure comparable scaling across features, particularly for models sensitive to feature magnitude such as LR and SVM. Within each fold of the LOSO framework, the standardization parameters (mean and standard deviation) were computed using only the training data and subsequently applied to both the training set and the held-out test subject. This procedure prevents information leakage and ensures a consistent and unbiased evaluation across folds.

In addition to the proposed feature set comprising Groups 1–3, an auxiliary baseline analysis was performed using only the conventional sleep architecture features (Group 1). This baseline was included to evaluate the contribution of the proposed transition dynamics and temporal evolution features. For this purpose, the same modeling framework, hyperparameter search space (Table 2), and LOSO cross-validation procedure were applied. Feature selection was conducted within each training fold by evaluating multiple subset sizes (k=6,8,10), and the best-performing configuration for each model was selected. This ensures a fair and consistent comparison between models trained on traditional sleep architecture features and those utilizing the full feature set.

### 2.5. Evaluation Metrics

To assess the performance of the proposed models in detecting MCI, several standard classification metrics were employed, including accuracy, sensitivity, specificity, balanced accuracy, and the area under the receiver operating characteristic curve (AUC-ROC). These metrics provide a comprehensive evaluation of model performance, particularly in the presence of class imbalance. In this study, the positive class corresponds to subjects with MCI, while the negative class represents healthy control subjects.

Let TP, TN, FP, and FN denote true positives, true negatives, false positives, and false negatives, respectively. Sensitivity (Recall) measures the proportion of correctly identified MCI subjects:

(3) $$\mathrm{Sensitivity}=\frac{TP}{TP+FN}$$

This metric reflects the model’s ability to detect MCI cases. Specificity quantifies the proportion of correctly identified healthy subjects:

(4) $$\mathrm{Specificity}=\frac{TN}{TN+FP}$$

This metric indicates how well the model avoids false alarms in healthy individuals. Accuracy represents the overall proportion of correct predictions:

(5) $$\mathrm{Accuracy}=\frac{TP+TN}{TP+TN+FP+FN}$$

However, accuracy alone can be misleading in imbalanced datasets. Therefore, Balanced Accuracy is also reported, which gives equal importance to both classes:

(6) $$\mathrm{Balanced}\;\mathrm{Accuracy}=\frac{\mathrm{Sensitivity}+\mathrm{Specificity}}{2}$$

In addition, the Area Under the Receiver Operating Characteristic Curve (AUC-ROC) was used to evaluate the model’s discriminative ability across different classification thresholds. The ROC curve plots the true positive rate (sensitivity) against the false positive rate (1−specificity), and the AUC summarizes the overall separability between MCI and healthy subjects. A higher AUC value indicates better model performance.

Given the relatively small sample size and potential class imbalance in the dataset, particular emphasis was placed on balanced accuracy and AUC-ROC, as these metrics provide a more reliable assessment of model generalization compared to accuracy alone.

### 2.6. Bootstrap-Based Variability Estimation

To ensure both clarity and robustness, a limited set of statistical procedures was employed, focusing on variability estimation and paired model comparison. The Leave-One-Subject-Out (LOSO) cross-validation framework provides a single aggregated estimate of model performance across all 53 subjects. However, due to the limited sample size, this point estimate does not capture the variability or statistical uncertainty of the evaluation metrics. To address this limitation, a stratified bootstrap procedure was employed using the subject-level LOSO predictions, which include the true labels, predicted labels, and predicted probabilities [31,32,33]. In this study, subjects were grouped into healthy controls (negative class) and MCI subjects (positive class), and sampling with replacement was performed independently within each class to preserve the original class distribution.

For each bootstrap iteration, resampling was performed at the subject level, where each subject represents a single independent observation characterized by a full-night hypnogram-derived feature set. Specifically, subjects were randomly sampled with replacement within each class (healthy and MCI), and the selected subjects were combined to form a bootstrap dataset of the same size as the original cohort. As a result, some subjects may appear multiple times in a given bootstrap sample, while others may be omitted, reflecting the variability inherent in the finite dataset. The evaluation metrics—accuracy, sensitivity, specificity, balanced accuracy, and AUC-ROC—were then computed for each resampled dataset using the corresponding subject-level predictions. This process was repeated 1000 times to generate empirical distributions for each metric, from which summary statistics including the mean, standard deviation, median, and 95% confidence intervals (2.5th and 97.5th percentiles) were obtained. This subject-level stratified bootstrap approach provides a robust estimation of performance variability and enhances the reliability of model comparisons. It should be noted that while bootstrap resampling provides an estimate of variability, it does not address limitations arising from small sample size or potential label uncertainty in the dataset.

### 2.7. Statistical Comparison of Models

To statistically compare the performance of models derived from novel and traditional feature sets, a paired bootstrap testing framework was employed [34,35]. In this study, the novel feature set incorporates Group 1, Group 2, and Group 3 features, representing sleep architecture, transition dynamics, and temporal evolution characteristics, respectively. In contrast, the traditional feature set includes only Group 1 (sleep architecture) features. For each model, subject-level predictions and associated probabilities obtained under the optimal hyperparameter configurations from grid search within the LOSO framework were used for subsequent analysis.

A paired comparison was performed by leveraging the subject-level independence of the dataset. For each bootstrap iteration b=1,…,B, a stratified sample of subjects was drawn with replacement while preserving the class distribution. Both the novel and traditional models were evaluated on the same resampled subject set, ensuring a paired comparison. For a given performance metric *M* (e.g., balanced accuracy, sensitivity, specificity), the difference between the two models was computed as:

(7) $$\Delta M^{\left(b\right)}=M_{\mathrm{novel}}^{\left(b\right)}-M_{\mathrm{traditional}}^{\left(b\right)}.$$

This procedure yields an empirical distribution of paired differences ${\left\{\Delta M^{\left(b\right)}\right\}}_{b=1}^{B}$, capturing the variability in performance differences across resampled datasets.

Statistical significance was assessed by testing the null hypothesis that there is no difference in performance between the two feature sets:

(8) $$H_{0}:E\left[\Delta M\right]=0.$$

A two-sided bootstrap hypothesis test was conducted by evaluating the proportion of bootstrap samples in which the sign of $\Delta M^{\left(b\right)}$ differed from the observed mean difference. The corresponding *p*-value was computed as twice the minimum of the proportions of non-positive and non-negative differences. In addition, 95% confidence intervals were obtained from the 2.5th and 97.5th percentiles of the empirical difference distribution.

This paired bootstrap framework enables direct comparison of performance metrics while accounting for subject-level variability and avoiding assumptions of normality. Unlike tests based solely on overall classification correctness, this approach allows for rigorous evaluation of class-sensitive metrics such as balanced accuracy and sensitivity, which are particularly relevant for assessing MCI detection performance [36]. The direction and magnitude of performance differences were interpreted based on the sign and distribution of $\Delta M^{\left(b\right)}$, providing both statistical and practical insight into the benefits of incorporating transition and temporal features.

## 3. Results

### 3.1. Feature Importance Results

To identify the most informative features for MCI classification, a RF–based feature importance analysis was performed within each fold of the LOSO framework. Specifically, for each training fold, an RF model was trained using all available features, and feature importance scores were computed based on the mean decrease in impurity. The top 10 features were then selected from each fold according to their importance rankings.

**Figure 3:**
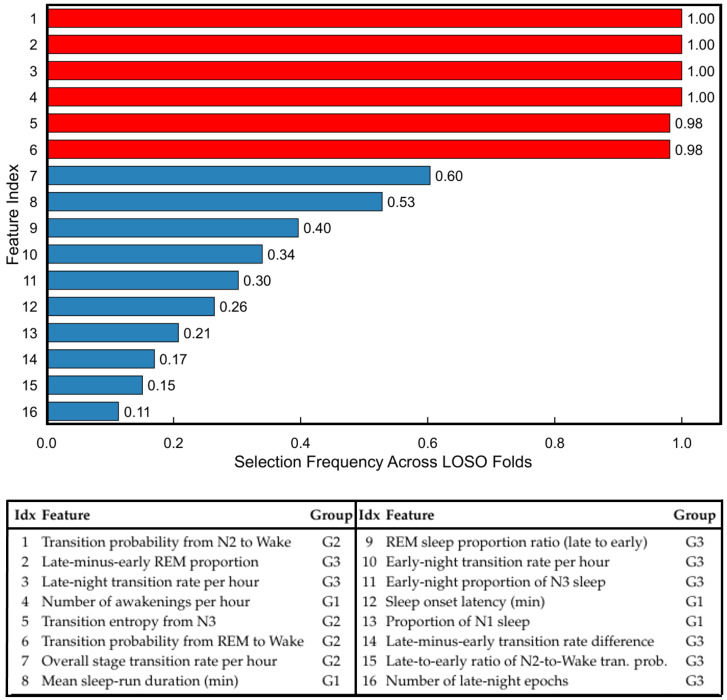
Selection frequency of the top 16 features across leave-one-subject-out (LOSO) folds. In each fold, a Random Forest–based feature importance analysis was used to select the top 10 features. The figure shows the 16 most frequently selected features across all folds. Six features (red) were consistently selected in nearly all folds, indicating their strong and stable contribution, while the remaining features (blue) were selected less consistently. Notably, five of the six most frequently selected features are from the novel feature groups proposed in this study. Feature indices correspond to the definitions provided in the embedded table.

To assess the stability and consistency of feature selection across subjects, the frequency with which each feature was selected among the top 10 was computed over all LOSO folds. Figure 3 summarizes the selection frequency of the 16 most frequently selected features. As shown, six features were consistently selected in nearly all folds, indicating their strong and stable contribution to the classification model. In contrast, the remaining features exhibited lower and more variable selection frequencies, suggesting comparatively weaker or subject-dependent relevance.

Notably, five of the six most consistently selected features belong to the novel feature groups proposed in this study, highlighting the effectiveness of the newly derived sleep dynamics descriptors. Furthermore, additional analysis using different selection thresholds (e.g., top 6 and top 8 features per fold) revealed a consistent pattern, where the same six features were repeatedly identified as the most important. This stability indicates that these features capture the dominant discriminative information in the dataset.

**Figure 4:**
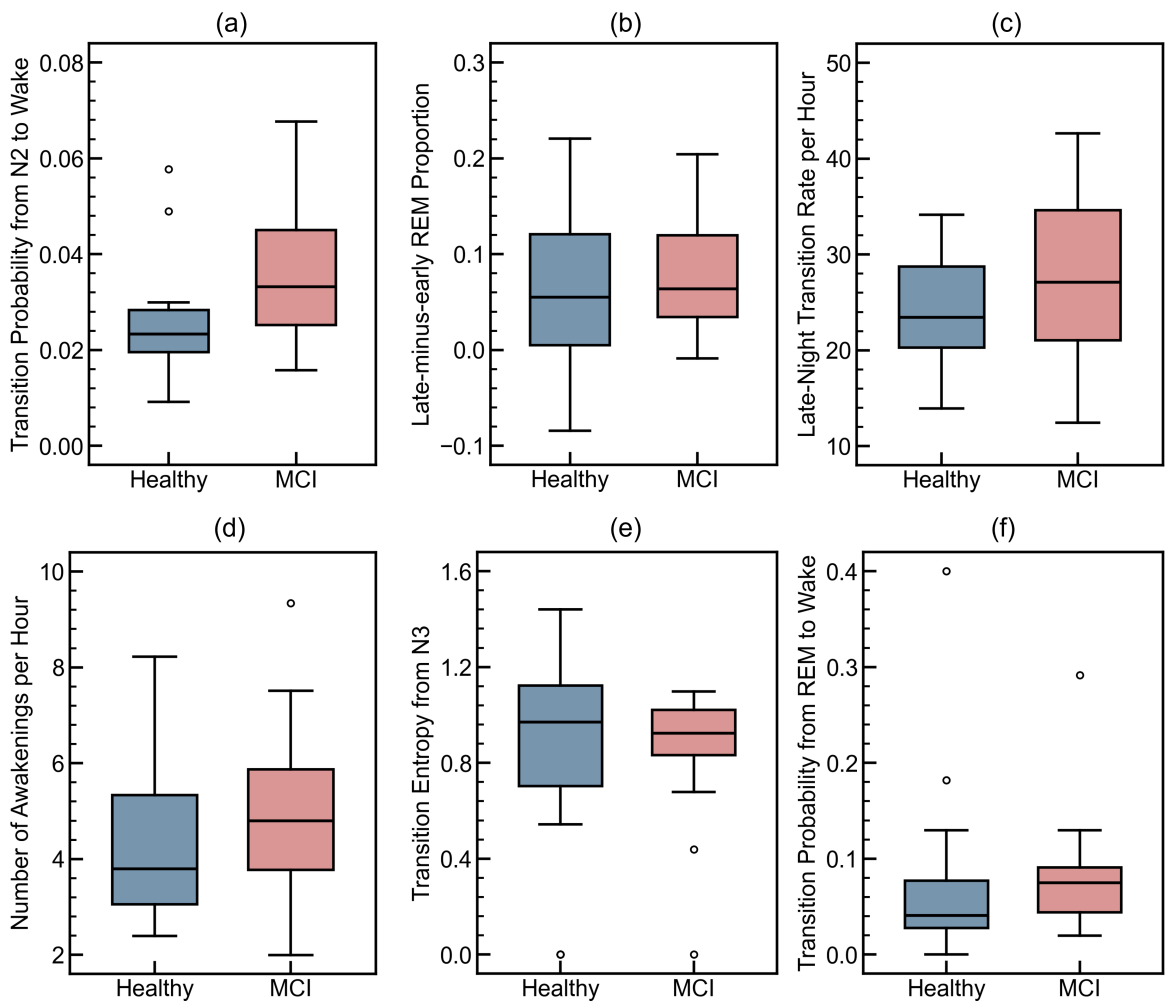
Boxplots of the six most frequently selected features for healthy and MCI subjects. (**a**) Transition probability from N2 to Wake, (**b**) late-minus-early REM proportion, (**c**) late-night transition rate per hour, (**d**) number of awakenings per hour, (**e**) transition entropy from N3, and (**f**) transition probability from REM to Wake. While some overlap exists between groups, consistent shifts in central tendency and distribution spread can be observed across multiple features, indicating their potential discriminative value.

Based on these observations, the top six features were selected for subsequent model development and hyperparameter tuning, ensuring a compact and robust feature set while minimizing redundancy. To further illustrate the distributional characteristics of these most informative features, Figure 4 presents boxplots comparing healthy and MCI subjects across the selected feature set.

As shown in Figure 4, several features exhibit noticeable shifts in distribution between healthy and MCI groups. In particular, features related to transition behavior and sleep fragmentation, such as transition rates and awakening frequency, tend to show higher median values and broader variability in the MCI group. Temporal evolution features, such as late-minus-early REM proportion, also demonstrate systematic differences between groups. Although overlap between the two groups is present, these trends highlight the discriminative patterns captured by the selected features.

### 3.2. Optimal Model Configurations and Performance of Proposed Features

To ensure a fair and consistent comparison, all models were optimized using grid search within the LOSO cross-validation framework, with balanced accuracy used as the selection criterion. A unified feature selection strategy was applied across all models, where the top-*k* features were selected within each training fold based on Random Forest importance rankings. Analysis of feature selection frequency (Figure 3) indicated that a small subset of features consistently dominated across folds, suggesting that the most informative sleep dynamics are captured by a compact representation. Based on this observation, k=6 features were selected as the optimal subset size for all models. The final hyperparameter configurations corresponding to these optimized settings are summarized in Table 3, and all reported results are obtained under the LOSO framework to ensure subject-independent evaluation.

**Figure 5:**
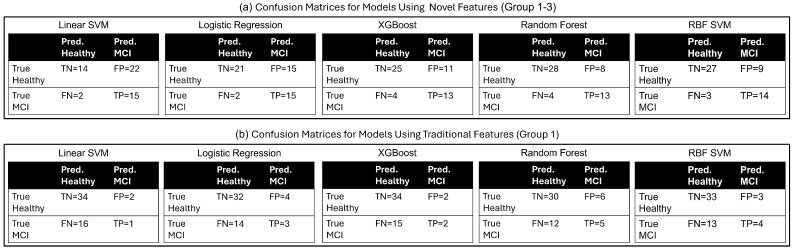
Confusion matrices of all models evaluated at their optimally tuned configurations obtained via grid search. (**a**) Results using the proposed feature set (Groups 1–3), which incorporates sleep architecture, transition dynamics, and temporal evolution features. (**b**) Results using only traditional sleep architecture features (Group 1). Each matrix summarizes classification outcomes for healthy and MCI subjects in terms of true negatives (TN), false positives (FP), false negatives (FN), and true positives (TP). Compared to the traditional feature set, the proposed feature set yields improved detection of MCI cases with a more balanced trade-off between sensitivity and specificity across models.

**Figure 6:**
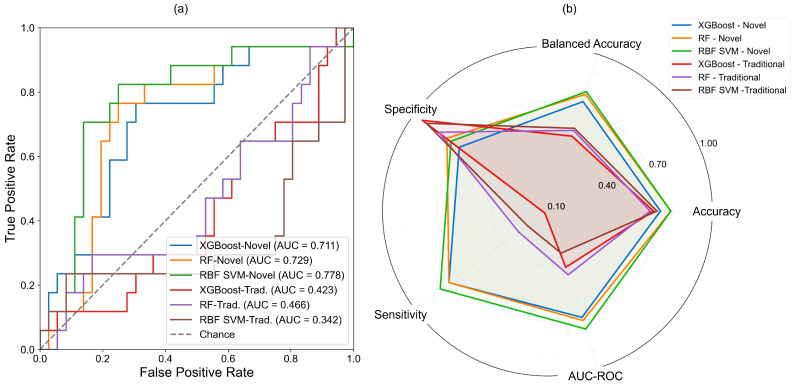
Comparison of the best-performing models (XGBoost, Random Forest, and RBF SVM) trained using the traditional feature set (Group 1) and the proposed feature set (Groups 1–3). Each model was evaluated at its optimally tuned configuration obtained via grid search. (**a**) Receiver operating characteristic (ROC) curves with corresponding AUC values, demonstrating the discriminative performance of each model. (**b**) Radar plot summarizing five key performance metrics: accuracy, balanced accuracy, sensitivity, specificity, and AUC-ROC. Across the three best performing models, the proposed feature set consistently outperforms the traditional feature set, yielding substantial improvements in balanced accuracy, sensitivity, and AUC-ROC, while maintaining competitive specificity.

Figure 5 presents the confusion matrices for all models evaluated using the proposed feature set, highlighting class-wise prediction behavior and revealing clear differences in how models balance detection of healthy and MCI subjects. Complementary performance comparisons are provided in Figure 6. The ROC curves (Figure 6a) demonstrate the discriminative capability of the best-performing models, with corresponding AUC values quantifying overall performance, while the radar plot (Figure 6b) summarizes key evaluation metrics, including accuracy, balanced accuracy, sensitivity, specificity, and AUC-ROC.

From a clinical perspective, false negatives (i.e., MCI subjects incorrectly classified as healthy) are of particular concern in a screening context, as they represent missed opportunities for early detection and intervention. While the proposed models demonstrate improved sensitivity compared to traditional feature sets, some false negatives remain, highlighting the need for further refinement to enhance detection reliability. Conversely, false positives may lead to additional follow-up evaluations, which, although less critical than missed cases, can increase clinical burden. Therefore, achieving an appropriate balance between sensitivity and specificity is essential for the practical deployment of such models in screening applications.

Among all models, the RBF SVM achieves the best overall performance, with the highest balanced accuracy (0.787) and AUC-ROC (0.778), indicating promising and consistent discrimination between classes. Random Forest follows closely, achieving comparable accuracy (0.774) and providing the most balanced trade-off between sensitivity (0.765) and specificity (0.778). XGBoost also demonstrates competitive performance, particularly with improved specificity compared to linear models, though with slightly reduced sensitivity. In contrast, linear models exhibit a pronounced imbalance between sensitivity and specificity. Both Linear SVM and Logistic Regression achieve high sensitivity (0.882), but their substantially lower specificity (0.389 and 0.583, respectively) leads to reduced balanced accuracy, indicating a tendency to over-predict the MCI class.

Overall, these results demonstrate that non-linear and ensemble-based models are better suited to capture the complex temporal structure of sleep-derived features, with RBF SVM, RF, and XGBoost consistently outperforming linear approaches in achieving more reliable and balanced MCI classification. While these results indicate meaningful discriminative capability, the achieved performance levels remain below those typically required for clinical screening applications, where higher sensitivity and specificity are necessary. Importantly, the consistent separation between healthy and MCI subjects across multiple models and evaluation metrics provides encouraging evidence supporting the central hypothesis of this study that hypnogram-derived sleep dynamics encode discriminative information relevant to cognitive impairment. The ability of a compact set of features (k=6) to achieve consistent classification further highlights the effectiveness of these features in capturing meaningful alterations in sleep architecture and transition behavior associated with MCI.

### 3.3. Comparison Between Novel and Traditional Feature Sets

To further evaluate the contribution of the proposed sleep dynamics features, we compare model performance using the full feature set (Group 1 + Group 2 + Group 3) against models trained using only conventional sleep architecture features (Group 1). To the best of our knowledge, no prior study has employed hypnogram-derived features within a machine learning framework for MCI classification. However, several studies have identified specific sleep architecture characteristics that are altered in MCI, which correspond to the features included in Group 1. In contrast, the transition dynamics and temporal evolution features (Groups 2 and 3) represent the primary novel contributions of this work.

For a fair comparison, the same modeling framework was applied to both feature sets. For the Group 1-only models, a separate grid search was conducted using the same hyperparameter ranges as in the combined-feature models. Feature selection was also performed within each LOSO fold, considering k=6,8, and 10 features, and the best-performing configuration for each model was selected. In most cases, the optimal models utilized k=8 or k=10 features. Feature importance analysis indicated that the most frequently selected features included number of awakenings per hour, proportion of N1 sleep, and wake after sleep onset (WASO), which are well-established markers in the literature. While these findings are consistent with existing studies, they appear insufficient for accurate classification of MCI when used alone.

Table 4 summarizes the performance comparison between the combined feature models and the Group 1-only models. Across all classifiers, the use of only Group 1 features results in substantially lower balanced accuracy and AUC-ROC. Notably, although specificity remains relatively high, sensitivity is significantly reduced, indicating a strong bias toward predicting healthy subjects and a failure to reliably detect MCI cases.

The confusion matrices for Group 1 models (Figure 5b) further illustrate this imbalance, with a large number of false negatives across all models. In contrast, the ROC curves and radar plot (Figure 6) demonstrate that incorporating the proposed sleep dynamics features leads to a more balanced and substantially improved performance across all evaluation metrics. In particular, the inclusion of Groups 2 and 3 features markedly improves sensitivity while maintaining strong specificity, enabling more reliable identification of MCI subjects.

These results highlight that while traditional sleep architecture features capture known physiological changes associated with MCI, they lack sufficient discriminative power for classification. The proposed transition dynamics and temporal evolution features provide complementary information that is critical for improving model performance and achieving robust MCI detection.

### 3.4. Bootstrap-Based Performance Variability Analysis

To further assess the robustness and stability of the proposed models, a bootstrap analysis was performed using repeated resampling of the subject-level predictions obtained under the LOSO framework using the procedure described in Section 2.6. For each model, performance metrics were recomputed across bootstrap samples, allowing estimation of variability around the observed performance.

**Figure 7:**
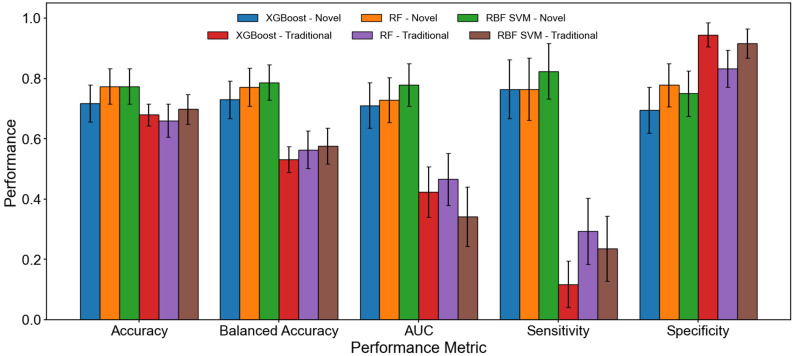
Performance comparison of the best-performing models (XGBoost, Random Forest, and RBF SVM) using the proposed feature set (Groups 1–3, denoted as Novel) and traditional sleep architecture features (Group 1). Bars represent the mean performance obtained from leave-one-subject-out cross-validation, while error bars indicate bootstrap-derived standard deviations. Across all evaluation metrics, models trained with the proposed feature set consistently achieve higher balanced accuracy, sensitivity, and AUC-ROC compared to their traditional counterparts. In contrast, traditional models exhibit high specificity but substantially reduced sensitivity, indicating a bias toward predicting healthy subjects.

Figure 7 presents a comparative visualization of model performance across key evaluation metrics, where bars denote the mean LOSO performance and error bars represent bootstrap-derived standard deviations. The figure includes both models trained using the proposed feature set (Groups 1–3) and those using only traditional sleep architecture features (Group 1), enabling a direct comparison of performance stability between the two approaches.

Consistent with the results obtained from the optimal LOSO configurations, RBF SVM demonstrates the strongest overall performance among all models using the proposed feature set, achieving the highest balanced accuracy and AUC-ROC with relatively low variability. Random Forest also exhibits stable and competitive performance, maintaining a well-balanced trade-off between sensitivity and specificity. XGBoost shows moderate variability but remains consistently better than linear models in terms of overall discrimination. In contrast, linear models exhibit greater variability and a pronounced imbalance in performance metrics. While Linear SVM and Logistic Regression maintain high sensitivity under the proposed feature set, their lower specificity and wider spread in bootstrap estimates indicate reduced reliability.

A clear contrast is observed when comparing models trained with traditional features. Although these models achieve high specificity with relatively low variability, their substantially reduced sensitivity and lower balanced accuracy indicate a strong bias toward predicting healthy subjects. This behavior is consistently observed across bootstrap samples, confirming that the limitation is not due to sampling variability but rather insufficient discriminative information in the feature space.

The corresponding numerical results, including mean LOSO performance and bootstrap-derived standard deviations, are summarized in Table 4. The consistency of performance across bootstrap samples further reinforces the reliability of the proposed approach. Importantly, the stability of classification performance—particularly for RBF SVM and Random Forest using the proposed feature set—confirms that the observed discrimination between healthy and MCI subjects is not driven by sampling variability, but reflects inherent structure in the hypnogram-derived sleep dynamics. These findings provide additional support for the central hypothesis of this work, demonstrating that sleep stage dynamics extracted from hypnograms can serve as effective and robust markers for MCI detection.

### 3.5. Paired Statistical Significance Testing

To further evaluate the performance differences between models trained with novel and traditional feature sets, a paired bootstrap statistical test was conducted as described in Section 2.6 and Section 2.7. The analysis was performed on the three best-performing models—XGBoost, RF, and RBF-SVM—using subject-level predictions obtained under their respective optimal configurations. For each performance metric, the difference between the novel and traditional models was computed across 1000 bootstrap iterations, and statistical significance was assessed using two-sided bootstrap hypothesis testing.

Table 5 summarizes the mean differences, 95% confidence intervals (CI), *p*-values, and significance outcomes for each model and evaluation metric. The reported mean differences represent the average paired improvement, defined as (Novel − Traditional), across bootstrap iterations, while the corresponding confidence intervals reflect the variability of these differences. A confidence interval that does not include zero indicates a statistically significant difference between the two models. Across all three models, balanced accuracy, AUC, and sensitivity demonstrate statistically significant improvements when using the proposed feature set. In particular, sensitivity shows consistently strong significance (p=0.002 for all models), indicating a substantial improvement in the detection of MCI subjects. Similarly, AUC exhibits notable gains, reflecting improved discrimination capability of the proposed features. Balanced accuracy, which accounts for class imbalance, also shows statistically significant improvements across all models, further confirming the effectiveness of the proposed approach in achieving more balanced classification performance.

In contrast, accuracy does not exhibit statistically significant differences for any of the evaluated models. This behavior is further explained by the specificity results. For the XGBoost model, specificity shows a statistically significant decrease (p<0.001), while for RF and RBF-SVM, specificity differences are not statistically significant. These observations indicate that traditional models tend to favor the majority class (healthy subjects), resulting in high specificity but poor sensitivity. The proposed feature set mitigates this bias by improving sensitivity, leading to more balanced classification performance without necessarily increasing overall accuracy.

Overall, the paired bootstrap analysis demonstrates that the proposed feature set provides statistically significant improvements in clinically relevant metrics, particularly sensitivity and balanced accuracy, while maintaining competitive overall performance. In addition to statistical significance, the observed differences correspond to meaningful effect sizes, indicating practical improvements in the detection of MCI subjects. These findings highlight the importance of incorporating transition dynamics and temporal evolution features for improved MCI detection. From a clinical perspective, the improvement in sensitivity is particularly relevant, as it reflects enhanced ability to identify individuals with cognitive impairment, although further validation with a much larger cohort is required to assess its effectiveness in real-world screening scenarios.

## 4. Discussion

### 4.1. Interpretation of Key Features

The feature importance analysis reveals a highly stable and consistent set of discriminative features for MCI classification. Notably, the top six features were selected in nearly all folds of the LOSO framework (i.e., across almost all 53 folds), indicating that they capture robust and subject-independent characteristics of sleep alterations associated with MCI. This consistency suggests that the identified features reflect meaningful physiological patterns rather than noise or subject-specific variability.

A key finding is the distribution of these dominant features across the defined feature groups. Among the six most consistently selected features, only one belongs to Group 1 (traditional sleep architecture), while three originate from Group 2 (transition dynamics) and two from Group 3 (temporal evolution). This strong representation of Group 2 and Group 3 features highlights the importance of the proposed sleep dynamics descriptors, with five of the six most informative features derived from the proposed feature groups introduced in this study.

These results emphasize the limitations of conventional approaches that rely primarily on static sleep architecture measures. While such features provide a general summary of sleep structure [37,38], they fail to capture the underlying temporal organization of sleep. In contrast, transition-based features characterize fragmentation and instability in sleep stage progression, while temporal evolution features capture intra-night changes such as altered REM distribution or disrupted slow-wave sleep patterns. These characteristics are consistent with well-established observations in the literature on sleep fragmentation and instability in MCI. The dominance of these features suggests that MCI-related sleep alterations are more strongly reflected in sleep dynamics than in overall stage proportions. Importantly, the contribution of the proposed feature set lies not in identifying new physiological mechanisms, but in providing a structured and quantitative representation of these established sleep behaviors that can be effectively leveraged for machine learning–based classification. In this context, the feature formulation is hypothesis-driven, grounded in prior physiological knowledge, while the identification of the most discriminative features and their relative importance is determined in a data-driven manner through the feature selection process.

Finally, the ability to achieve strong and consistent feature selection across different thresholds (e.g., top 6, top 8, and top 10 per fold) suggests the presence of stable discriminative patterns in the data. However, given the limited dataset size, the repeated selection of a small subset of features across folds may also reflect dataset-specific structure or implicit bias, rather than purely generalizable patterns. The resulting compact feature set of six features enables the development of lightweight and interpretable models, which are well-suited for wearable and home-based monitoring applications. Overall, these findings demonstrate that modeling sleep as a dynamic and temporally evolving process provides a more informative framework for MCI detection than traditional static descriptors.

### 4.2. Clinical and Neurobiological Interpretation

The distributions of the most frequently selected features, as shown in Figure 4, provide insight into the physiological significance of the identified sleep dynamics. A consistent pattern across multiple features is the tendency for MCI subjects to exhibit higher transition activity and increased sleep fragmentation. For example, features such as late-night transition rate (Figure 4c), number of awakenings per hour (Figure 4d), and transition probabilities from N2 to Wake and REM to Wake (Figure 4a,f) all show elevated median values and broader distributions in the MCI group. These patterns are consistent with well-established observations of disrupted sleep continuity in individuals with cognitive impairment and may reflect reduced stability of thalamocortical circuits responsible for maintaining consolidated sleep states [37,39].

In addition to fragmentation-related features, temporal evolution characteristics also exhibit systematic differences between groups. The late-minus-early REM proportion (Figure 4b) indicates altered intra-night REM dynamics in MCI subjects, suggesting deviations from the normal progression of sleep architecture across the night. Such alterations may be associated with early cholinergic dysfunction, which plays a key role in REM sleep regulation [40]. Furthermore, the transition entropy from N3 (Figure 4e) reflects irregularity in deep sleep transitions, which may be linked to impaired slow-wave sleep organization. Reduced stability in slow-wave sleep has been associated with diminished glymphatic clearance and increased accumulation of neurotoxic proteins, providing a plausible connection between sleep disruption and neurodegenerative processes [41,42].

It is important to emphasize that the proposed framework does not establish causal relationships between these features and specific neurobiological mechanisms. Rather, the observed patterns are consistent with existing literature on sleep fragmentation, REM disruption, and slow-wave sleep alterations in MCI. These interpretations provide a physiological context for the discriminative features identified by the model and support the relevance of hypnogram-derived sleep dynamics as potential markers of early cognitive decline.

### 4.3. Performance of Models with Proposed Features

The comparative evaluation of multiple machine learning models highlights the promising discriminative capacity of hypnogram-derived sleep features for MCI classification. Overall, non-linear and ensemble-based models consistently outperform linear approaches, indicating that the relationship between sleep dynamics and cognitive impairment is inherently non-linear. Among all models, the RBF SVM achieved the best performance, with the highest balanced accuracy (0.787) and AUC-ROC (0.778). Random Forest and XGBoost followed closely, with Random Forest providing the most balanced trade-off between sensitivity (0.765) and specificity (0.778), and XGBoost showing competitive performance with improved specificity. The consistent results across these fundamentally different model families suggest that the observed classification performance is driven by meaningful structure in the data rather than model-specific behavior.

In contrast, linear models (Linear SVM and Logistic Regression) exhibited a clear imbalance between sensitivity and specificity. While both achieved high sensitivity (0.882), their substantially lower specificity led to reduced balanced accuracy, indicating a tendency to over-predict the MCI class. This limitation reflects the inability of linear models to adequately capture the complex relationships present in the proposed sleep dynamics features [43]. Additionally, during model optimization, certain small values of the regularization parameter (*C*) in the RBF SVM resulted in near-perfect classification. However, these occurrences were isolated and inconsistent across neighboring parameter values, indicating overfitting rather than true performance improvement [44,45]. Consequently, only stable and generalizable configurations were considered in the final evaluation.

It is important to interpret these results within the context of the study design. This work represents a proof-of-concept approach based solely on hypnogram-derived features, without relying on raw EEG signals, imaging data, or expert-driven annotations beyond standard sleep staging. Despite this simplified representation, the models achieve balanced accuracies approaching 0.78, demonstrating that meaningful biomarkers of cognitive impairment are embedded within sleep stage dynamics. However, it is important to emphasize that the achieved performance levels, while encouraging, remain below those typically required for clinical screening applications, where substantially higher sensitivity and specificity are necessary to ensure reliable detection.

Furthermore, these findings must be interpreted with caution due to limitations in the underlying dataset. The MASS SS1 dataset includes 36 healthy subjects and 17 MCI subjects, of which only 15 are clinically confirmed, while two were classified as borderline MCI patients based on updated diagnostic criteria. As these subjects could not be excluded, all were treated as MCI, introducing potential label ambiguity and a degree of label noise. In addition, the relatively small cohort size and class imbalance may contribute to variability in model performance across classifiers. While the use of LOSO validation ensures subject-independent evaluation and bootstrap resampling provides insight into performance variability, these approaches cannot fully compensate for limitations related to sample size and cohort heterogeneity. In particular, the combination of feature selection, hyperparameter tuning, and model evaluation within the same dataset, even under a nested LOSO framework, may introduce implicit overfitting that is not fully captured by internal validation. Consequently, the reported results should be considered as an internal estimate, and the generalizability of the proposed framework to broader clinical populations remains to be established through validation on larger and independent cohorts.

Taken together, these results should be viewed as demonstrating the feasibility of using hypnogram-derived sleep dynamics for MCI classification rather than establishing a clinically deployable screening solution. The gap between proof-of-concept classification and clinically actionable screening remains substantial and requires further methodological refinement and large-scale validation.

Finally, the consistent performance across multiple models and the ability to achieve strong results using only six features further reinforce the robustness of the proposed approach. At the same time, this consistency should be interpreted in the context of the limited dataset size, where repeated use of the same cohort may introduce implicit bias despite careful validation procedures. This compact feature set captures the essential characteristics of MCI-related sleep alterations while enabling the development of lightweight and interpretable models. Overall, these findings provide encouraging evidence that hypnogram-derived sleep dynamics constitute a viable and informative domain for MCI detection, supporting the central hypothesis of this study and offering a foundation for future improvements using larger and more refined datasets.

### 4.4. Comparison of Novel and Traditional Feature Sets

The comparison between models trained using only conventional sleep architecture features (Group 1) and those using the combined feature set (Groups 1–3) provides direct evidence of the contribution of the proposed sleep dynamics features. Existing literature on sleep and MCI has largely focused on static architectural descriptors, such as stage proportions and fragmentation measures, which are represented in Group 1. However, our results demonstrate that these features alone are insufficient for reliable classification. Across all models, Group 1-only configurations yield substantially lower balanced accuracy and AUC, indicating limited discriminative power when used in isolation.

A key observation is the strong imbalance in Group 1-only models, characterized by very low sensitivity and high specificity. This indicates a tendency to predict subjects as healthy, resulting in a large number of missed MCI cases (false negatives). Given the dataset composition (36 healthy and 17 MCI subjects), a trivial classifier that predicts all subjects as healthy would achieve an accuracy of approximately 0.679 and a balanced accuracy of 0.5. Notably, the performance of several Group 1-only models approaches this baseline, confirming that traditional sleep architecture features alone do not provide sufficient information to distinguish MCI subjects.

The observed shift in performance between the traditional and proposed feature sets, characterized by improved sensitivity and a moderate increase in false positives, warrants clarification. Importantly, the feature extraction process is independent of model training and is applied uniformly to all subjects prior to cross-validation. Within the LOSO framework, feature selection and hyperparameter optimization are performed strictly within each training fold, ensuring that no information from the test subject is used during model development. Therefore, the observed performance differences do not arise from parallel optimization or data leakage, but rather reflect the differing characteristics of the feature sets.

Specifically, traditional sleep architecture features tend to emphasize dominant patterns associated with the majority class (healthy subjects), leading to higher specificity but reduced sensitivity. In contrast, the proposed transition and temporal features capture more subtle and dynamic alterations in sleep behavior that are more sensitive to MCI-related changes. As a result, the model becomes more effective at identifying MCI subjects, at the cost of an increased number of false positives. This trade-off is commonly observed in imbalanced classification problems and reflects a shift toward improved detection of the minority class rather than an artifact of the modeling pipeline.

In contrast, the inclusion of transition dynamics and temporal evolution features (Groups 2 and 3) leads to a clear and consistent improvement across all models. The combined feature models achieve higher balanced accuracy and AUC while maintaining a more appropriate trade-off between sensitivity and specificity. In particular, sensitivity is substantially improved, enabling more reliable identification of MCI subjects without sacrificing specificity. This demonstrates that the proposed features capture complementary and clinically relevant information related to the temporal organization of sleep that is not reflected in static measures.

These findings strongly support the two primary contributions of this study. First, the results demonstrate that a compact set of novel features derived from sleep stage transitions and intra-night dynamics provides meaningful and discriminative information for MCI detection. Second, the results show that these features can be effectively utilized within lightweight machine learning models to achieve robust classification performance. The ability to significantly outperform traditional feature sets using only a small number of interpretable features highlights the potential of this approach for practical, scalable, and non-invasive monitoring of cognitive decline using sleep data.

### 4.5. Robustness and Statistical Significance Analysis

The bootstrap analysis provides an estimate of the variability of model performance under resampling of the available data. The relatively small standard deviations and reasonably narrow confidence intervals observed for the top-performing models (RBF SVM and Random Forest) suggest consistent performance across bootstrap samples. This is particularly important given the limited sample size, as it indicates that the observed performance is not highly sensitive to sampling variability within the dataset.

An important observation is that RBF SVM and Random Forest demonstrate superior and stable performance, while XGBoost remains competitive and clearly outperforms linear models. In contrast, linear models exhibit larger variability and persistently imbalanced behavior, reinforcing the conclusion that simple linear decision boundaries are insufficient for capturing the structure of hypnogram-derived features [43,46]. The consistency of these findings across both LOSO and bootstrap analyses strengthens the validity of the overall modeling framework.

The paired bootstrap statistical analysis provides additional insight into the comparative behavior of the top-performing models at the subject level. While RBF-SVM, Random Forest, and XGBoost exhibit improvements in several aggregate performance metrics when using the proposed feature set, these improvements are not uniform across all metrics. In particular, overall accuracy does not show statistically significant differences for any of the models, with mean differences remaining modest (e.g., 4.2% for XGBoost, 9.8% for Random Forest, and 6.0% for RBF-SVM), indicating that the total number of correctly classified subjects remains comparable between the novel and traditional feature representations.

However, a more detailed examination reveals that statistically significant improvements are consistently observed in balanced accuracy, AUC, and sensitivity across all models. Balanced accuracy improves by approximately 19.7–20.3% across the three models, reflecting a substantial enhancement in class-balanced performance. Similarly, AUC shows notable gains, with mean increases of 26.7% for Random Forest, 29.0% for XGBoost, and 42.8% for RBF-SVM, indicating improved discrimination capability. Most importantly, sensitivity exhibits the largest improvements, increasing by 47.3% to 64.9%, demonstrating a significantly enhanced ability to detect MCI subjects.

In contrast, specificity does not show consistent improvement and, in some cases, decreases (e.g., a reduction of 24.3% for XGBoost), indicating that traditional feature sets tend to favor the majority class (healthy subjects). This trade-off highlights that the proposed feature set shifts the decision boundary toward better detection of the minority class, leading to more balanced classification performance. Overall, these findings demonstrate that the performance gains associated with the proposed feature set arise from improved detection of the positive class rather than a general increase in overall accuracy. This emphasizes the importance of evaluating class-sensitive metrics in imbalanced clinical classification tasks, where improvements in sensitivity are often more meaningful than changes in aggregate accuracy.

Taken together, these findings highlight that the discriminative information lies primarily in the extracted features rather than in the choice of a specific model. The fact that multiple, structurally different models achieve comparable and stable performance suggests that the proposed sleep dynamics features encode consistent and meaningful patterns associated with MCI. However, this consistency should be interpreted with caution given the limited dataset size and the repeated use of the same cohort across model development and evaluation. This robustness across models and validation strategies provides encouraging internal evidence and further supports the central hypothesis of this study within the context of this dataset. Nevertheless, external validation on independent cohorts is required to confirm the reliability and generalizability of the proposed approach.

### 4.6. Benchmarking with Existing Machine Learning Approaches

Table 6 presents a survey of representative machine learning-based approaches for MCI detection across different modalities and datasets. Direct comparison of performance metrics across these studies is inherently challenging due to the absence of standardized public datasets and substantial variability in subject populations, data acquisition protocols, and evaluation strategies. Many prior works rely on proprietary datasets with differing class distributions and sample sizes. In contrast, this study utilizes the publicly available MASS SS1 dataset, which, although relatively small (17 MCI and 36 healthy subjects), satisfies the requirements for sleep-based analysis and is comparable in scale to several datasets used in existing literature.

From a performance standpoint, the proposed method does not aim to achieve the highest reported accuracy but instead introduces a novel framework based solely on hypnogram-derived sleep dynamics that can also be obtained by processing EEG data. To the best of our knowledge, this feature representation has not been previously explored for MCI classification within a machine learning setting. As a proof-of-concept study, there remains significant potential for further improvement through larger datasets and refined feature engineering. Nevertheless, the achieved performance (balanced accuracy up to 0.787) is competitive with several existing machine learning approaches and comparable to, or exceeding, a number of reported methods, despite relying on a simplified and interpretable feature set.

Most studies summarized in Table 6 rely on raw EEG signals or multimodal inputs, often requiring complex preprocessing or additional clinical data. While deep learning approaches have been proposed, this summary intentionally focuses on lightweight machine learning models to support practical deployment. For example, the CEEDNet model [52] reports an accuracy of 0.747 on a large EEG dataset, while its Random Forest counterpart performs substantially worse (0.466), indicating that increased model complexity does not necessarily translate to improved performance. In contrast, the proposed approach achieves comparable results using compact features and computationally efficient models derived from sleep stage sequences. Furthermore, the proposed hypnogram-based features can be combined with complementary features extracted directly from EEG signals for MCI classification, which may further improve performance beyond that achieved using either feature set independently.

Among the surveyed studies, only a limited number of works [27,28] explicitly explore wearable EEG-based solutions. These approaches demonstrate promising performance using devices such as the MUSE EEG headband and incorporate additional modalities, including handwriting tasks and virtual reality-based assessments. While these findings indicate the potential for at-home MCI detection, they still rely on controlled experimental setups, active subject participation, and, in many cases, supervision by trained personnel during data collection. In contrast, the proposed hypnogram-based framework is inherently passive, wearable-friendly, and fully automatable. By leveraging sleep stage sequences that can be obtained from existing wearable sleep monitoring devices and automated sleep staging algorithms [22], the system eliminates the need for active tasks, specialized equipment, or technician involvement. Once integrated into a wearable platform, the proposed method has the potential to enable continuous, non-invasive overnight monitoring, where MCI risk can be assessed automatically following a full-night sleep recording. Such a framework is potentially suitable for future large-scale, at-home monitoring applications, where repeated measurements can provide early warning signals and prompt further clinical evaluation if necessary. However, a substantial gap remains between proof-of-concept classification and clinically validated screening systems, and further validation on larger and diverse populations is required before such deployment can be realized.

Overall, these findings highlight that while many existing approaches achieve strong performance using complex or multimodal data, the proposed method offers a unique combination of simplicity, interpretability, and practical deployability. This positions the framework as a promising step toward scalable and real-world MCI screening using sleep-based biomarkers.

## 5. Limitations

This study has several important limitations that should be considered when interpreting the results. First, the dataset size is relatively small, comprising 53 subjects with 17 MCI cases, including two borderline cases, which introduces potential label uncertainty and may contribute to variability in model performance. In addition, no independent validation cohort was available; therefore, the LOSO cross-validation framework provides only an internal estimate of performance. Consequently, the generalizability of the proposed framework to broader and more diverse populations remains to be established.

Furthermore, the feature selection and model optimization processes were performed within the same dataset, raising the possibility of implicit overfitting despite the use of fold-wise procedures to minimize data leakage. The repeated use of a limited cohort for feature selection, hyperparameter tuning, and evaluation may introduce dataset-specific bias that is not fully captured by internal validation. In addition, the analysis relies on expert-annotated polysomnographic sleep stages rather than automatically derived sleep staging, which may limit direct applicability to real-world wearable systems where additional variability is expected.

Finally, while the proposed features are grounded in established sleep physiology, their biological interpretation remains indirect and requires further validation using multimodal data. Although the proposed framework demonstrates promising classification performance, the achieved accuracy and AUC values remain below those typically required for clinical screening applications. Therefore, further validation on larger and independent cohorts, along with continued methodological refinement, is necessary before the proposed approach can be considered for clinical or large-scale deployment.

## 6. Conclusions

In this study, we proposed a novel and lightweight framework for MCI detection based on hypnogram-derived sleep stage dynamics. The results demonstrate that a compact set of physiologically interpretable features capturing transition behavior and intra-night temporal evolution can effectively distinguish between healthy and MCI subjects. Across multiple machine learning models and validation strategies, consistent and robust performance was observed, with non-linear models achieving balanced accuracies approaching 0.78. Importantly, the proposed features significantly outperformed conventional sleep architecture descriptors, highlighting the value of modeling sleep as a dynamic and temporally evolving process. These findings provide encouraging evidence that sleep stage dynamics encode meaningful information related to early cognitive impairment and support the central hypothesis of this study. However, further validation is required before these results can be translated into clinical screening applications. Overall, the proposed framework provides a foundation for scalable, non-invasive screening approaches for the diagnosis of MCI.

Future work will focus on extending this framework to larger and more diverse datasets to further validate its generalizability. Additionally, while the current study utilizes expert-annotated sleep stages, an important next step is the integration of automated sleep staging algorithms with the proposed MCI classification model within a unified pipeline. Moreover, the overall accuracy of the MCI classification with the proposed features will likely be improved with the addition of features for MCI classification extracted from the EEG data. This will enable a fully automated, end-to-end system for real-time cognitive risk assessment using wearable sleep monitoring devices. Such advancements have the potential to facilitate continuous, at-home screening for early detection of cognitive decline, ultimately supporting timely intervention and improved clinical outcomes.

## Figures and Tables

**Table 1:** Summary of extracted sleep features grouped by physiological interpretation.

Feature Group	Description	No. of Features
Group 1: Sleep Architecture	Stage proportions (W, N1, N2, N3, REM), sleep efficiency, sleep onset and REM latency, wake after sleep onset (WASO), awakenings per hour, mean stage durations, and stage entropy	12
Group 2: Sleep Transition Dynamics (Novel)	Transition rate, full transition probability matrix (all stage-to-stage transitions), self-transition probabilities, and transition entropy measures	45
Group 3: Temporal Evolution —Early vs. Late Night (Novel)	Early vs. late differences and ratios of selected architecture and transition features, including stage proportions, fragmentation metrics, and key transition probabilities	23
**Total**		**80**

**Table 2:** Machine learning models, tuned hyperparameters, and search ranges used in grid search.

Used Model	Tuned Parameters	Search Range
Logistic Regression	Regularization strength (C) Penalty type Solver	C∈[0.01,0.1,1,10,100] Penalty ∈ {L1, L2} Solver ∈ {liblinear, saga}
Random Forest	Number of trees Maximum depth Minimum samples split Minimum samples leaf Max features	n_estimators∈[100,200,300] max_depth∈[3,5,7,None] min_samples_split∈[2,5,10] min_samples_leaf∈[1,2,4] max_features∈ {sqrt, log2}
Linear SVM	Regularization parameter (C) Loss	C∈[0.01,0.1,1,10,100] Loss ∈ {hinge, squared hinge}
RBF SVM	Regularization parameter (C) Kernel coefficient (γ)	C∈[0.1,1,10,100] γ∈ {scale, 0.01, 0.001}
XGBoost	Learning rate Max depth Number of trees Subsample Colsample	learning_rate∈[0.03,0.05,0.1] max_depth∈[2,3,4] n_estimators∈[100,200,300] subsample∈[0.7,0.8,1.0] colsample_bytree∈[0.7,0.8,1.0]

**Table 3:** Best-performing hyperparameter values selected from grid search.

Used Model	Parameter	Selected Value
Logistic Regression	Regularization strength (C) Penalty type Solver	C=1.0 Penalty = L2 Solver = liblinear
Random Forest	Number of trees Maximum depth Minimum samples split Minimum samples leaf Max features	n_estimators=100 max_depth=2 min_samples_split=2 min_samples_leaf=1 max_features=sqrt
Linear SVM	Regularization parameter (C) Loss	C=1.0 Loss = squared hinge
RBF SVM	Regularization parameter (C) Kernel coefficient (γ)	C=10.0 γ=scale
XGBoost	Learning rate Max depth Number of trees Subsample Colsample	learning_rate=0.03 max_depth=2 n_estimators=100 subsample=0.8 colsample_bytree=0.8

**Table 4:** Performance comparison between models trained using the proposed novel feature set (Groups 1–3) and traditional sleep architecture features (Group 1). Reported values represent the mean performance obtained from leave-one-subject-out cross-validation, with bootstrap-derived standard deviations shown in brackets. Across all models, the proposed feature set yields consistently higher balanced accuracy and AUC, along with substantially improved sensitivity, indicating more reliable detection of MCI subjects while maintaining competitive specificity.

Feature Set	Model	Acc.	Bal. Acc.	AUC	Sens.	Spec.
Novel	Linear SVM	0.547 [±0.060]	0.636 [±0.055]	0.556 [±0.082]	0.882 [±0.077]	0.389 [±0.081]
	LR	0.679 [±0.062]	0.733 [±0.056]	0.657 [±0.081]	0.882 [±0.077]	0.583 [±0.085]
	XGBoost	0.717 [±0.061]	0.730 [±0.063]	0.711 [±0.075]	0.765 [±0.098]	0.694 [±0.076]
	RF	0.774 [±0.059]	0.771 [±0.063]	0.729 [±0.074]	0.765 [±0.103]	0.778 [±0.071]
	RBF SVM	0.774 [±0.059]	0.787 [±0.059]	0.778 [±0.070]	0.824 [±0.092]	0.750 [±0.075]
Traditional	Linear SVM	0.660 [±0.018]	0.502 [±0.013]	0.111 [±0.051]	0.059 [±0.021]	0.944 [±0.027]
	LR	0.660 [±0.047]	0.533 [±0.054]	0.423 [±0.085]	0.176 [±0.095]	0.889 [±0.053]
	XGBoost	0.679 [±0.036]	0.531 [±0.043]	0.423 [±0.084]	0.118 [±0.077]	0.944 [±0.040]
	RF	0.660 [±0.054]	0.564 [±0.062]	0.466 [±0.087]	0.294 [±0.109]	0.833 [±0.061]
	RBF SVM	0.698 [±0.048]	0.576 [±0.060]	0.342 [±0.098]	0.235 [±0.107]	0.917 [±0.048]

**Table 5:** Paired bootstrap statistical comparison between the three best performing models trained with novel and traditional feature sets. Differences are computed as (Novel − Traditional). The reported 95% confidence intervals (CI) correspond to the empirical distribution of these paired differences across bootstrap iterations. A CI that does not include zero indicates a statistically significant difference between the two models.

Model	Metric	Mean Difference	95% CI	*p*-Value	Significant
XGBoost	Accuracy	0.042	[−0.113, 0.189]	0.644	No
	Balanced Accuracy	0.203	[0.013, 0.372]	0.034	Yes
	AUC	0.290	[0.075, 0.482]	0.012	Yes
	Sensitivity	0.649	[0.294, 0.941]	0.002	Yes
	Specificity	−0.243	[−0.389, −0.083]	0.000	Yes
RF	Accuracy	0.098	[−0.057, 0.226]	0.222	No
	Balanced Accuracy	0.197	[0.022, 0.353]	0.026	Yes
	AUC	0.267	[0.087, 0.443]	0.012	Yes
	Sensitivity	0.473	[0.176, 0.765]	0.002	Yes
	Specificity	−0.079	[−0.250, 0.083]	0.418	No
RBF-SVM	Accuracy	0.060	[−0.094, 0.208]	0.532	No
	Balanced Accuracy	0.200	[0.020, 0.367]	0.030	Yes
	AUC	0.428	[0.176, 0.636]	0.002	Yes
	Sensitivity	0.588	[0.294, 0.824]	0.002	Yes
	Specificity	−0.189	[−0.389, 0.000]	0.080	No

**Table 6:** Survey of Machine Learning-Based Approaches for Mild Cognitive Impairment Detection Across Modalities and Datasets.

Ref.	Year	Data (MCI/HC)	Modality	Method	Acc.	Bal. Acc.	Sens.	Spec.	AUC	Wearable	Auto.
EEG-Based Approaches											
[29]	2023	Own (42/51)	EEG	Gentle Boost	0.810	0.810	0.820	0.800	–	**✗**	∼
[47]	2023	Own (61/59)	EEG	Soft Voting	0.823	–	–	–	–	**✗**	∼
[48]	2024	Own (18/16)	EEG	SVM	0.673	0.653	0.997	0.309	–	**✗**	∼
[49]	2020	Own (13/27)	EEG	FC & Graph	0.775	0.713	0.538	0.889	–	**✗**	∼
[50]	2023	Own (47/402)	EEG	eLORETA-ICA	0.782	–	–	–	0.849	**✗**	∼
[51]	2019	Own (8/15)	EEG	SVM	0.879	0.899	0.848	0.950	–	**✗**	∼
[52]	2023	Own (417/459)	EEG	CEEDNet	0.747	–	–	–	–	**✗**	**✗**
[52]	2023	Own (417/459)	EEG	RF	0.466	–	–	–	–	**✗**	∼
Multimodal and Alternative Approaches											
[30]	2023	Own (189/246)	EEG + CSF + APOE	SVM	0.767	–	–	–	–	**✗**	**✗**
[27]	2023	Own (40/39)	Wearable EEG	RBF-SVM	0.861	–	–	–	–	✔	✔
[27]	2023	Own (40/39)	Wearable EEG	RF	0.797	–	–	–	–	✔	✔
[27]	2023	Own (40/39)	Handwriting	RBF-SVM	0.835	–	–	–	–	✔	✔
[27]	2023	Own (40/39)	Handwriting	RF	0.785	–	–	–	–	✔	✔
[28]	2023	Own (44/42)	Wearable EEG + VR	RBF-SVM	0.840	–	–	–	–	✔	✔
[28]	2023	Own (44/42)	Wearable EEG + VR	RF	0.790	–	–	–	–	✔	✔
[53]	2018	Own (48/38)	Speech	SVM / RF	0.714	0.708	0.750	0.667	0.708	**✗**	**✗**
Proposed Hypnogram-Based Approach											
**Our**	2026	MASS SS1 (17/36)	Hypnogram	RF	0.774	0.771	0.765	0.778	0.729	✔	✔
**Our**	2026	MASS SS1 (17/36)	Hypnogram	RBF-SVM	0.774	0.787	0.824	0.750	0.778	✔	✔

Note: “Own” indicates that the dataset was collected and used by the authors of the respective study and is not publicly available. The symbol ✔ indicates that the criterion is satisfied, **✗** indicates that it is not satisfied, ∼ indicates partial or limited feasibility, and – indicates that the corresponding value was not reported in the cited study. Wearable compatibility and automation feasibility were qualitatively assessed based on data acquisition requirements and processing complexity reported in each study. Reported performance values are not directly comparable across studies due to differences in datasets, subject populations, modalities, and evaluation protocols Acronyms—Acc: Accuracy; Bal. Acc.: Balanced accuracy; Sens: Sensitivity (Recall) Spec: Specificity; Auto.: Automation feasibility; CSF: Cerebrospinal Fluid; APOE: Apolipoprotein E genotype; eLORETA-ICA: exact low resolution brain electromagnetic tomography-independent component analysis; CEEDNet: CAUEEG end-to-end deep neural network; VR: Virtual reality.

## Data Availability

The data used in this study are derived from the Montreal Archive of Sleep Studies (MASS), specifically the SS1 subset. The MASS dataset is publicly available at https://borealisdata.ca (accessed on 30 January 2025) upon registration and agreement with the data usage terms defined by the providers. Access to certain components of the dataset, including the MCI subject data, may be restricted and must be obtained separately from the dataset owners in accordance with their data sharing policies.

## Abbreviations

The following abbreviations are used in this manuscript:

MCI	Mild Cognitive Impairment
MASS	Montreal Archive of Sleep Studies
EEG	Electroencephalography
LR	Logistic regression
RF	Random forest
XGBoost	Extreme gradient boosting
SVM	Support vector machine
RBF-SVM	Radial basis function support vector machine
ROC-AUC	Receiver operating characteristic–area under the curve
MMSE	Mini-mental state examination
MoCA	Montreal Cognitive Assessment
MRI	Magnetic resonance imaging
LOSO	Leave-one-subject-out
AASM	American academy of sleep medicine
NC	Not classified
REM	Rapid eye movement
WASO	Wake after sleep onset
